# Validity of shoe-type inertial measurement units for Parkinson’s disease patients during treadmill walking

**DOI:** 10.1186/s12984-018-0384-9

**Published:** 2018-05-15

**Authors:** Myeounggon Lee, Changhong Youm, Jeanhong Jeon, Sang-Myung Cheon, Hwayoung Park

**Affiliations:** 1 Biomechanics Laboratory, College of Health Sciences, Dong-A University, Hadan 2-dong, Saha-gu, Busan, Republic of Korea; 2 Department of Health Care and Science, College of Health Sciences, Dong-A University, 37 Nakdong-Daero 550 beon-gil, Hadan 2-dong, Saha-gu, Busan, Republic of Korea; 3 Department of Neurology, School of Medicine, Dong-A University, Dongdaesin-dong 3-ga, Seo-gu, Busan, Republic of Korea

**Keywords:** Parkinson’s disease, Inertial measurement unit, Spatiotemporal parameter, Gait, Validity

## Abstract

**Background:**

When examining participants with pathologies, a shoe-type inertial measurement unit (IMU) system with sensors mounted on both the left and right outsoles may be more useful for analysis and provide better stability for the sensor positions than previous methods using a single IMU sensor or attached to the lower back and a foot. However, there have been few validity analyses of shoe-type IMU systems versus reference systems for patients with Parkinson’s disease (PD) walking continuously with a steady-state gait in a single direction. Therefore, the purpose of this study is to assess the validity of the shoe-type IMU system versus a 3D motion capture system for patients with PD during 1 min of continuous walking on a treadmill.

**Methods:**

Seventeen participants with PD successfully walked on a treadmill for 1 min. The shoe-type IMU system and a motion capture system comprising nine infrared cameras were used to collect the treadmill walking data with participants moving at their own preferred speeds. All participants took anti-parkinsonian medication at least 3 h before the treadmill walk. An intraclass correlation coefficient analysis and the associated 95% confidence intervals were used to evaluate the validity of the resultant linear acceleration and spatiotemporal parameters for the IMU and motion capture systems.

**Results:**

The resultant linear accelerations, cadence, left step length, right step length, left step time, and right step time showed excellent agreement between the shoe-type IMU and motion capture systems.

**Conclusions:**

The shoe-type IMU system provides reliable data and can be an alternative measurement tool for objective gait analysis of patients with PD in a clinical environment.

**Electronic supplementary material:**

The online version of this article (10.1186/s12984-018-0384-9) contains supplementary material, which is available to authorized users.

## Background

Gait analysis is a robust method for investigating many health-related factors and has been utilized to determine overall health and predict the cognitive decline, risk of falling, quality of life, and lifespan of patients [1]. Patients with Parkinson’s disease (PD), which is a progressive neurodegenerative disorder, experience gait disturbances such as a shuffling gait, reduced step length, reduced gait speed, and delayed gait initiation [2, 3]. These factors may increase the risk of falling [4]. Therefore, gait analysis of patients with PD is being actively studied to investigate the progression of neurodegenerative diseases [5].

In general, the gait of patients with PD is analyzed with objective evaluation methods using measurement devices [6]. Motion capture systems and instrumented walkway systems are considered the gold standard for gait analysis and are usually employed because they provide precise measurements for spatiotemporal and kinematic variables [5, 7]. However, these systems tend to be expensive and require a huge amount of laboratory space, extended post-processing, and skilled technicians. Furthermore, they can generally only capture a small number of consecutive steps in a small capture volume, which can limit the averaged step data, and it is doubtful whether the collected data are similar to natural walking patterns in daily life [8]. Thus, they may be difficult to utilize in a clinical environment [3, 7–11]. Consequently, various researchers have proposed inertial measurement unit (IMU) systems as an alternative method for gait analysis [3, 7, 9, 11–13]. An IMU system comprises tri-axial accelerometers and gyroscopes. It is a wearable device that can be miniaturized [10, 12, 14]. IMU systems have the advantages of being relatively inexpensive, small, lightweight, and requiring a relatively small amount of laboratory space compared to conventional systems [10, 11, 13]. IMU systems can evaluate objective measurements of spatiotemporal gait parameters quickly and easily in a clinical environment [7].

Previous studies have conducted validity analyses of IMU systems versus motion capture systems by using healthy adults [11, 12, 14, 15] and reported excellent agreement during gait-related tasks between spatiotemporal parameters [11, 12, 14] and linear accelerations [15]. Several researchers have performed validity analyses of IMU systems versus reference systems by using a motion capture system and instrumented walkway system with participants with pathologies [5, 7, 13, 16, 17], and good to excellent agreement during gait-related tasks was found for the spatiotemporal parameters [5, 7, 13, 16, 17]. However, most previous studies conducted validity analyses by using a single IMU sensor [5, 7, 16, 17]. A single sensor attached to the lower back is used in the inverted pendulum model, where walking is carried out in a straight line at a constant pace [5]. However, participants with PD have sustained gait disturbances such as a decreased step length and shuffling step, which may lead to estimation errors such as longer or shorter time variables, more or fewer gait cycles, and an incorrect step length [5, 13]. Moreover, participants with PD have higher accelerations along the anteroposterior, mediolateral, and vertical axes than healthy controls, and these results may be due to the difficulties that participants with PD have with walking smoothly [18]. Thus, increased acceleration may cause overestimation of the vertical displacement of the COM, which may lead to overestimation of the step length [7]. Trojaniello et al. [16] suggested that using IMU sensors attached to each lower limb bilaterally when analyzing participants with pathologies with a gait disturbance may increase the detection accuracy of gait-related events such as the heel strike (HS) and toe off (TO), and it may provide precise and accurate data for participants with PD [5, 13, 16].

The position of the IMU sensor is also important for increased data accuracy during gait analysis. Previous studies attached the IMU sensor to the skin above the fourth to fifth lumbar vertebra [5, 7], on the left and right lateral malleolus [13], or to the shoe [11, 17]. However, a sensor attached to the body may measure skin motion artifacts as well as dynamic acceleration due to a tilted position from conditions such as lumbar lordosis and sensor position inaccuracy, which may in turn influence the calculation accuracy of the spatiotemporal variables [8]. Joo et al. [12] reported that a shoe-type IMU system and motion capture system indicated excellent agreement for the cadence (*ICC* = 0.998) and step length (*ICC* = 0.970) of 1 min of treadmill walking by healthy young and older adults. They suggested that a shoe-type IMU system mounted on both the left and right outsoles may be a more effective and objective way to detect gait-related events and evaluate spatiotemporal gait parameters [10, 12]. However, few studies have assessed the validity of shoe-type IMU systems versus motion capture systems during continuous walking by participants with PD. Furthermore, most previous studies conducted validity analyses along a walkway with limited space and used repeated measurement methods such as multiple walking trials [5, 13, 16] or continued walking and turning at a point until the time limit [7, 17]. These methods may have limited applicability because of the averaging of several trials of data, and it remains to be seen whether the averaged data can represented the real walking patterns of participants [8]. Thus, a validity analysis of participants with PD walking continuously with a steady-state gait in a single direction may provide meaningful results. The purpose of this study was to assess the validity of the shoe-type IMU system versus a 3D motion capture system for patients with PD during 1 min of continuous treadmill walking. The working hypothesis was that the shoe-type IMU system with two sensors and the motion capture system would indicate excellent agreement for the resultant linear acceleration, cadence, step length, and step time.

## Methods

### Participants

An a priori power analysis was conducted to determine the minimum sufficient sample size for an effect size of 0.5, power of 80%, and significance of 0.05. Based on this analysis, 26 participants were required. Thirty patients with PD were recruited who met the UK Brain Bank criteria for PD diagnosis [19] and various Hoehn and Yahr (H&Y) stages of severity [20].

**Fig. 1:**
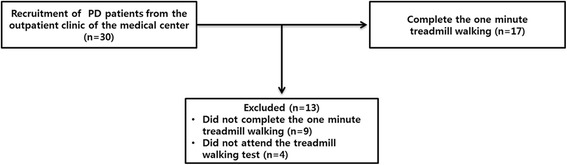
Flow diagram illustrating inclusion criteria for participants

The patients were from the outpatient clinic of a medical center. The criteria for inclusion in the study were as follows: (a) diagnosed with idiopathic PD, (b) H&Y stage 1–3, (c) taking anti-parkinsonian medication, (d) a Mini-Mental State Exam (MMSE) score of more than 24 points, and (e) no medical history of orthopedic surgery, neurosurgery, and neurophysiology within 6 months prior to the study. Nine participants were excluded owing to them not successfully completing the 1-min treadmill walking activity, and four participants did not attend the treadmill walking test. Consequently, 17 participants with PD successfully carried out 1 min of treadmill walking in this study (Fig. 1, Table 1).

**Table 1:** Clinical and demographic characteristics

Characteristics	Participants with PD (Males = 8; Females = 9)
Age (yr)	64.6 ± 7.4
Height (cm)	158.7 ± 9.4
Body mass (kg)	64.5 ± 9.7
BMI (kg/m^2^)	25.6 ± 3.5
Self-preferred walking speed (km/h)	1.42 ± 0.78
MDS-UPDRS total (score)	68.0 ± 14.2
MDS-UPDRS part III (score)	41.5 ± 11.0
H&Y stage	2.3 ± 0.4
MMSE (score)	27.8 ± 2.2
Duration of disease (yr)	5.9 ± 3.0
Levodopa equivalent dose (mg)	643.24 ± 306.55

*m ± sd* mean and standard deviation, *BMI* Body mass index, *H&Y* Hoehn and Yahr, *MMSE* Mini-mental state examination, *MDS-UPDRS* Modified Movement Disorder Society version of the unified Parkinson’s disease rating scale, *PD* Parkinson’s disease

The severity of PD was assessed in terms of the H&Y stage, which was charted from unilateral involvement of the disability (stage 1), bilateral involvement of the disability without impairment of balance (stage 2), bilateral involvement of the disability with postural instability (stage 3), severe disability but still able to walk and stand without assistance (stage 4), and wheelchair-bound or bedridden unless aided (stage 5) [20]. The modified Movement Disorder Society version of the Unified Parkinson’s Disease Rating Scale (MDS-UPDRS) has four evaluation parts. Part III of the MDS-UPDRS, which ranges from 0 (no motor symptoms) to 132 (severe motor symptoms) [5], was applied in the motor examination. All participants read and signed an informed consent form approved by the institutional review board of Dong-A University (IRB number: 2–104,709-AB-N-01-201,606-HR-025-04).

### Instrumentation

**Fig. 2:**
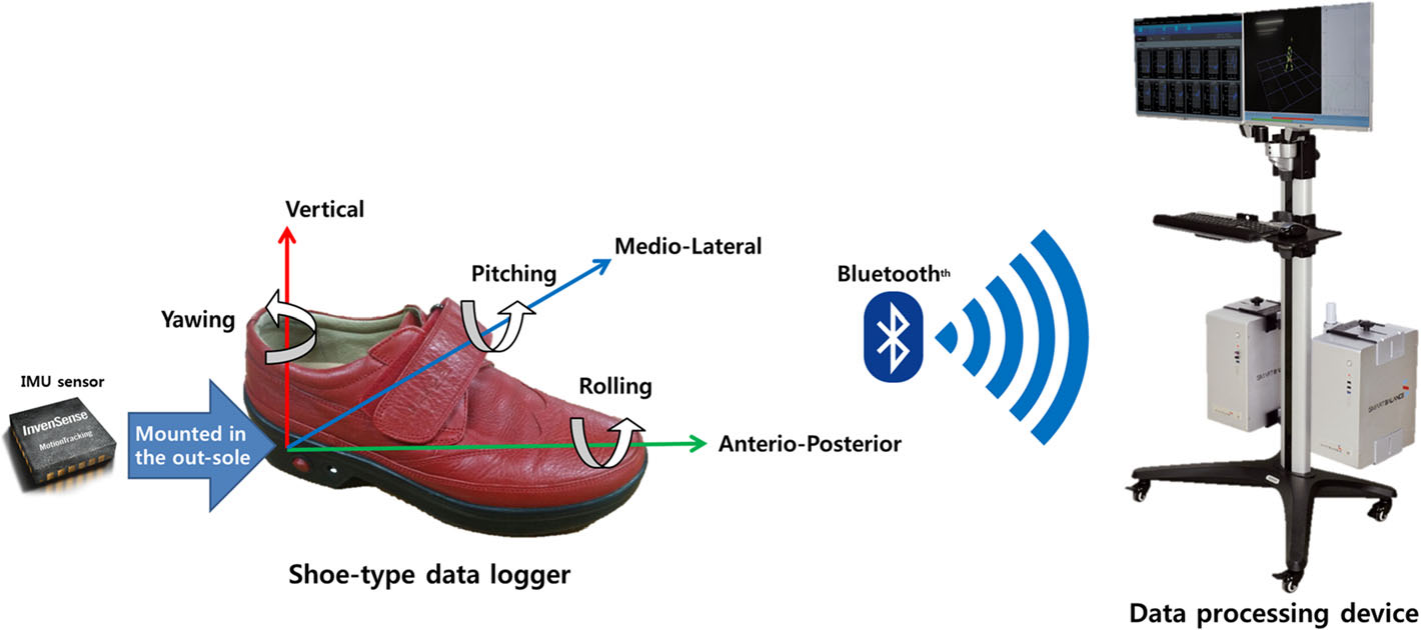
Local coordinate system of the shoe-type IMU system

Shoe-type IMU sensor-based gait analysis systems (DynaStab™, JEIOS, South Korea) consisting of shoe-type data loggers (Smart Balance® SB-1, JEIOS, South Korea) and a data acquisition system were utilized. The shoe-type data logger included an IMU sensor (IMU-3000™, InvenSense, USA) that could measure tri-axial acceleration (up to ±6 *g*) and tri-axial angular velocities (up to ±500° s^− 1^) along three orthogonal axes [10, 12]. The IMU sensors were installed in both outsoles of the shoes, and the data were transmitted wirelessly to a data acquisition system via Bluetooth®. The shoe sizes were adapted to individuals, and a range of shoe sizes was available from 225 mm to 280 mm. The local coordinate system of the IMU sensors was established with anteroposterior, mediolateral, and vertical directions (Fig. 2).

**Fig. 3:**
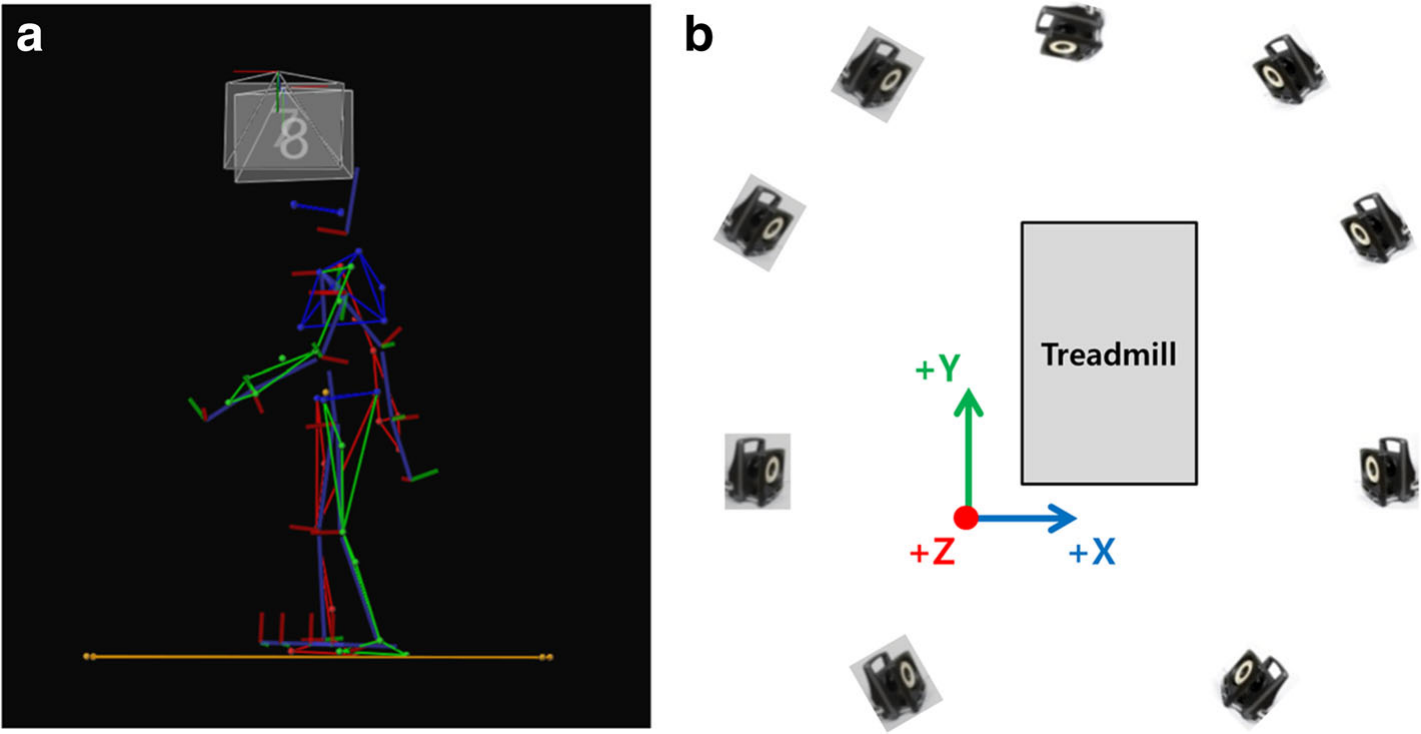
Motion capture system. **a** Treadmill walking. **b** Experimental setup

The motion capture system was composed of nine infrared cameras (MX-T10, Vicon, UK) and one acquisition system (MX-Giganet, Vicon, UK) for treadmill walking. The orientation of the global coordinate system was set behind the left side of the treadmill along the mediolateral (X-axis), anteroposterior (Y-axis), and vertical directions. The motion capture volume was set at 2.0 m (width) × 2.5 m (length) × 2.5 m (height) (Fig. 3). The speed of the belt on the treadmill (LGT7700M, LEXCO, South Korea) could be controlled from 0.5 km/h to 20 km/h in increments of 0.1 km/h. During data collection, both the IMU systems and motion capture systems sampled data at 100 Hz.

### Experimental procedures

All participants took anti-parkinsonian medication at least 3 h prior to treadmill walking. Before the treadmill walking, all participants with PD were assessed by a PD specialist in terms of the MDS-UPDRS, H&Y stage, MMSE, and duration of disease. All participants performed a warmup protocol comprising a stretching program and treadmill walking practice at their self-preferred speed for 10 min with a professional exercise trainer. The self-preferred speed was defined as the speed at which a participant was able to move with a stable gait without any support while walking on a treadmill. The treadmill speed was gradually increased until the self-preferred speed of the participants was reached.

After each PD patient completed the warmup procedure, their bodies were measured to obtain the values for their models. The body height and weight were measured with a stadiometer and body weight scale. The widths of the shoulders, elbows, wrists, and knees and the ankle and hand thicknesses were measured with a caliper. The length of the leg was measured from the anterior superior iliac spine to the medial malleolus with a tape measure. After the body measurements were completed, all participants were asked to wear Lycra shirts and shorts, and IMU sensors were mounted in the shoes. Thirty-nine spherical reflective markers, each with a 14 mm diameter, were attached to the participants in accordance with the plug-in gait full-body model (Vicon, Oxford, UK) [21]. All markers were attached to bony landmarks on the participants with double-sided tape and Kinesio tape for stability.

In the treadmill walking test, all participants were first asked to walk on the treadmill at their self-preferred speed. The participants walked approximately 30–60 s from the start of the gait in order to maintain a steady-state gait at their self-preferred speed. The steady-state gait was defined as that when the participant maintained a stable gait movement at a constant speed. The treadmill walking test may be more useful for collecting steady-state gait data than the over ground walking test. The latter allows the acceleration and deceleration of the participants during the gait initiation and termination phases; thus, these were excluded from the analysis [22]. The treadmill walking test was concluded to be more suitable than the over ground walking test because this study was mainly focused on the validity of the shoe-type IMU system versus a 3D motion capture system. When the participant exhibited a steady-state gait, an operator collected the treadmill walking data for 1 min.

### Data analysis

**Fig. 4:**
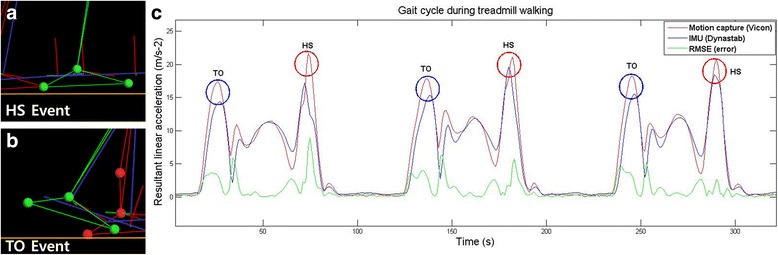
Data from a representative PD participant. The figure shows the resultant linear accelerations during treadmill walking with the shoe-type IMU system (DynaStab) and motion capture system (Vicon). The red line shows the motion capture system data; the blue line shows the IMU system data; the green line shows the root mean square error (RMSE) between the IMU and motion capture systems; the red circles show the timing of HS; and the blue circles show the timing of TO. **a** heel strike event. **b** toe off event

The IMU system and motion capture system data were filtered by using a second-order Butterworth low-pass filter with a cutoff frequency of 10 Hz [10, 12]. The data were simultaneously collected from both measurement systems and synchronized based on the timing of the HS and TO by using MATLAB® (2012a, MathWorks Inc., USA) (Fig. 4).

The variables of the IMU system were compared to those of the motion capture system for the resultant linear accelerations and spatiotemporal parameters during the 1-min treadmill walking test. The resultant linear accelerations from the IMU system were calculated as the net accelerations along the X, Y, and Z axes for the left and right shoes individually (Eq. 1). For the motion capture system, the double differential of the heel marker’s position along the X, Y, and Z axes was used to calculate the accelerations. The marker of the heel was located closest to the IMU sensor in order to minimize the phase difference between the two systems. The resultant linear accelerations were calculated using the net accelerations along the X, Y, and Z axes for the left and right shoes individually (Eq. 1).

1 $$ \mathrm{Resultant}\ \mathrm{linear}\ \mathrm{acceleration}=\sqrt{x_{acce}^2+{y}_{acce}^2+{z}_{acce}^2} $$

The error between the IMU system (a_IMU_(R)) and motion capture system (a_MC_(R)) was derived by averaging the root mean square error (RMSE) over the total signal for the resultant linear acceleration [7] (Eq. 2). The %RMSE was defined as the averaged ratio of the RMSE value [23].

2 $$ \mathrm{RMSE}=\sqrt{\frac{1}{N}\sum \limits_{k=1}^N{\left({a}_{IMU}(R)-{a}_{MC}(R)\right)}^2} $$

The gait-related events for both the IMU and motion capture systems were defined such that the HS was when the resultant linear acceleration reached the maximum value, and the TO was when the resultant linear acceleration reached the second maximum value during a gait cycle (Fig. 4). The spatiotemporal parameters for both the IMU system and motion capture system were calculated as follows. (a) The cadence (step/min) was calculated as the total number of steps during 1 min. (b) The step length was defined as the product of the walking speed and step time. (c) The step time was defined as the period between the HS of one foot to the subsequent HS of the contralateral foot.

### Statistical analysis

All statistical analyses were performed by using SPSS for Windows (version 20.0, SPSS Inc., Chicago, IL). The Shapiro–Wilk test was used to determine whether the data had a normal distribution. An intraclass correlation coefficient (*ICC* (2,1); two-way random single measures) analysis and the associated 95% confidence intervals (CIs) were used to assess the validity of the resultant linear acceleration and spatiotemporal parameters of the IMU system to that of the motion capture system. The limits of agreement (LOA) was calculated according to the Bland–Altman plots to show the differences between two systems [24]. The statistical significance level was set at 0.05.

## Results

**Fig. 5:**
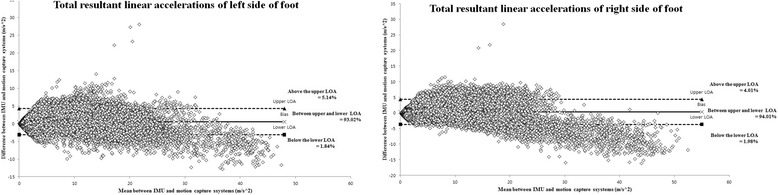
Bland–Altman plots of the all participants. The figure shows the resultant linear accelerations for both the left and right shoes during 1 min of treadmill walking

**Fig. 6:**
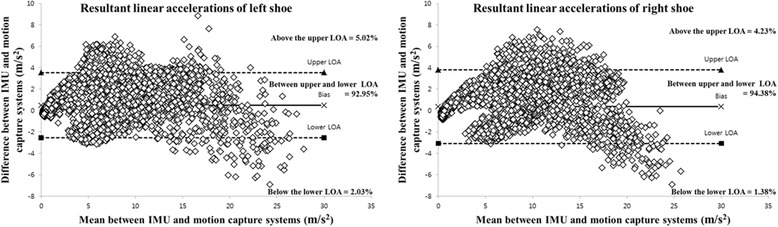
Bland–Altman plots of the representative participant. The figure shows the resultant linear accelerations for both the left and right shoes during 1 min of treadmill walking

In the validity analysis for the shoe-type IMU system and motion capture system, the resultant linear accelerations for the 17 participants with PD indicated excellent agreement for both the left (*ICC* = 0.973, 95% CI = 0.973–0.974, *p* < 0.001) and right (*ICC* = 0.971, 95% CI = 0.971–0.972, *p* < 0.001) shoes during the 1-min treadmill walking. The resultant linear accelerations for each participant indicated excellent agreement for both the left (*ICC* range = 0.961–0.985) and right (*ICC* range = 0.944–0.982) shoes (Table 2). Figures 5 and 6 show the difference between the two systems for the resultant linear accelerations of the left and right shoes during 1-min treadmill walking by all PD participants and representative participant, respectively. The largest difference was between the upper and lower LOA for both shoes (range = 93.02–94.01%).

**Table 2:** Results of the validity analysis. The table presents the resultant linear acceleration for each participant with PD when measured with the shoe-type IMU system versus the motion capture system

	Left shoe				Right shoe			
	*ICC* (2,1)	95% CI of *ICC* (min, max)	RMSE (m/s^2^)	Percent RMSE (%)	*ICC* (2,1)	95% CI of *ICC* (min, max)	RMSE (m/s^2^)	Percent RMSE (%)
Participant 1	0.982^*^	0.981, 0.983	1.630	6.05	0.978^*^	0.977, 0.979	1.791	7.59
Participant 2	0.983^*^	0.982, 0.984	2.896	6.67	0.979^*^	0.978, 0.980	2.997	7.07
Participant 3	0.970^*^	0.968, 0.971	1.366	6.13	0.957^*^	0.954, 0.959	2.050	7.33
Participant 4	0.963^*^	0.961, 0.964	1.602	7.25	0.955^*^	0.953, 0.958	1.785	7.18
Participant 5	0.961^*^	0.959, 0.963	1.850	6.83	0.980^*^	0.978, 0.981	1.842	5.13
Participant 6	0.985^*^	0.985, 0.986	1.368	4.78	0.982^*^	0.981, 0.983	1.389	4.78
Participant 7	0.962^*^	0.960, 0.963	1.221	6.71	0.957^*^	0.954, 0.959	1.524	8.97
Participant 8	0.980^*^	0.979, 0.981	1.689	5.58	0.970^*^	0.968, 0.971	1.909	6.46
Participant 9	0.982^*^	0.981, 0.983	1.649	6.32	0.968^*^	0.967, 0.970	1.884	7.21
Participant 10	0.956^*^	0.954, 0.958	2.642	10.11	0.966^*^	0.964, 0.967	2.117	7.91
Participant 11	0.971^*^	0.969, 0.972	1.867	6.83	0.955^*^	0.953, 0.957	2.055	7.59
Participant 12	0.961^*^	0.959, 0.963	2.618	10.44	0.951^*^	0.949, 0.954	2.483	8.16
Participant 13	0.967^*^	0.965, 0.968	2.175	6.12	0.964^*^	0.963, 0.966	1.546	4.67
Participant 14	0.973^*^	0.971, 0.974	1.156	4.62	0.976^*^	0.975, 0.977	1.207	5.50
Participant 15	0.977^*^	0.976, 0.978	2.564	7.45	0.958^*^	0.956, 0.960	1.855	6.32
Participant 16	0.964^*^	0.962, 0.966	2.362	8.21	0.944^*^	0.941, 0.947	2.132	9.65
Participant 17	0.964^*^	0.962, 0.966	3.254	6.62	0.970^*^	0.969, 0.972	3.582	7.49
M ± SD	0.971 ± 0.009		1.994 ± 0.629	6.87 ± 1.56	0.965 ± 0.011		2.009 ± 0.579	7.00 ± 1.39

*: results of the correlation analysis, *p* < 0.001; *CI* confidence interval, *ICC* intraclass correlation coefficient; *RMSE* root mean square error

**Fig. 7:**
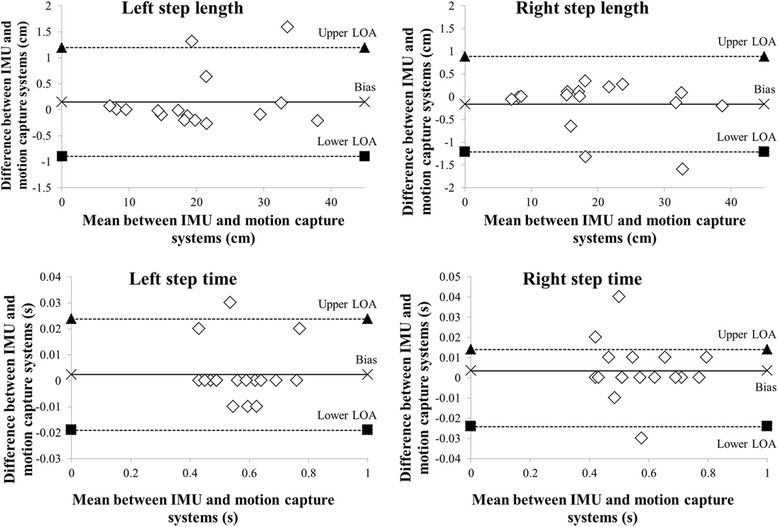
Bland–Altman plots of the all participants. The figure shows the spatiotemporal parameters for both the left and right shoes during 1 min of treadmill walking

**Fig. 8:**
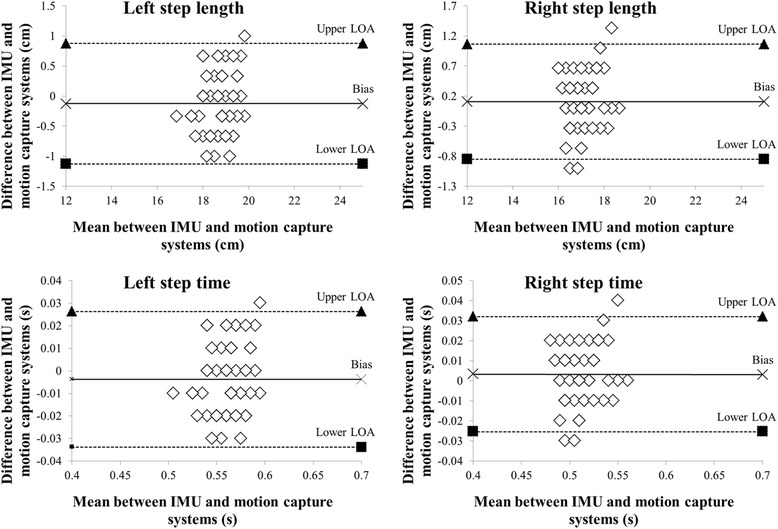
Bland–Altman plots of the representative participant. The figure shows the spatiotemporal parameters for both the left and right shoes during 1 min of treadmill walking

All spatiotemporal parameters were normally distributed. The results for the cadence (*ICC* = 1.000, *p* < 0.001), left step length (*ICC* = 0.990, *p* < 0.001), right step length (*ICC* = 0.999, *p* < 0.001), left step time (*ICC* = 0.993, *p* < 0.001), and right step time (*ICC* = 0.993, *p* < 0.001) showed excellent agreement (Table 3). The difference between the two systems for all spatiotemporal parameters was illustrated by Bland–Altman plots (Figs. 7, 8). The spatiotemporal parameters for each participant indicated moderate to excellent agreement for both the left (*ICC* range = 0.734–0.997) and right (*ICC* range = 0.731–0.997) shoes (Tables 4 and 5).

**Table 3:** Validity analysis of the spatiotemporal parameters. The table presents the results for the shoe-type IMU system and motion capture system

	IMU system	Motion capture system	*ICC* (2,1)	95% CI of *ICC min, max*	
				IMU system	Motion capture system
Cadence (steps/min)	108.24e (ste	108.24e (ste	1.000^*^	97.91–118.56	97.91–118.56
Left step length (cm)	22.11step l	22.26step l	0.990^*^	15.99–28.24	16.10–28.43
Right step length (cm)	22.53 step	22.37 step	0.999^*^	15.68–29.39	15.55–29.19
Left step time (s)	0.57 step	0.57 step	0.993^*^	0.50–0.63	0.51–0.63
Right step time (s)	0.57 t ste	0.57 t ste	0.993^*^	0.50–0.63	0.51–0.63

m.63: mean and standard deviation; *: results of the correlation analysis, *p* < 0.001; *CI* confidence interval, *ICC* intracorrelation coefficient, *IMU* inertial measurement unit

**Table 4:** Results of the validity analysis of the left shoe. The table presents the spatiotemporal parameters for each participant with PD when measured with the shoe-type IMU system versus the motion capture system

Left shoe						
	Step length *(ICC)*	95% CI *min*, *max* (cm)		Step time *(ICC)*	95% CI *min*, *max* (s)	
		IMU system	Motion capture system		IMU system	Motion capture system
Participant 1	0.734^*^	18.57–18.92	18.42–18.82	0.734^*^	0.56–0.57	0.55–0.56
Participant 2	0.906^*^	32.42–33.07	34.02–34.65	0.906^*^	0.42–0.43	0.44–0.45
Participant 3	0.983^*^	7.94–8.32	7.96–8.32	0.983^*^	0.48–0.50	0.48–0.50
Participant 4	0.920^*^	17.16–17.48	17.15–17.46	0.921^*^	0.62–0.63	0.62–0.63
Participant 5	0.997^*^	20.17–21.91	21.00–22.57	0.997^*^	0.73–0.79	0.76–0.81
Participant 6	0.994^*^	14.31–15.33	14.19–15.25	0.994^*^	0.74–0.79	0.73–0.78
Participant 7	0.972^*^	6.93–7.25	7.00–7.32	0.972^*^	0.42–0.44	0.42–0.44
Participant 8	0.902^*^	18.43–18.88	19.75–20.21	0.902^*^	0.51–0.52	0.55–0.56
Participant 9	0.735^*^	14.31–14.57	14.21–14.45	0.735^*^	0.47–0.48	0.47–0.47
Participant 10	0.758^*^	32.35–32.72	32.49–32.83	0.758^*^	0.58–0.59	0.58–0.59
Participant 11	0.928^*^	18.14–18.47	17.94–18.28	0.928^*^	0.54–0.55	0.54–0.55
Participant 12	0.919^*^	52.98–53.86	53.00–53.84	0.919^*^	0.68–0.69	0.68–0.69
Participant 13	0.932^*^	19.70–20.10	19.49–19.90	0.932^*^	0.64–0.66	0.64–0.65
Participant 14	0.941^*^	9.44–9.61	9.43–9.60	0.941^*^	0.49–0.49	0.49–0.49
Participant 15	0.986^*^	21.22–22.12	20.95–21.86	0.986^*^	0.59–0.61	0.58–0.61
Participant 16	0.982^*^	29.15–29.88	29.05–29.79	0.982^*^	0.62–0.63	0.62–0.63
Participant 17	0.982^*^	37.54–38.69	37.33–38.49	0.982^*^	0.45–0.46	0.45–0.46

*: results of the correlation analysis, *p* < 0.001 *CI* confidence interval, *ICC* intraclass correlation coefficient, *IMU* inertial measurement unit

**Table 5:** Results of the validity analysis of the right shoe. The table presents the spatiotemporal parameters for each participant with PD when measured with the shoe-type IMU system versus the motion capture system

Right shoe						
	Step length *(ICC)*	95% CI *min*, *max* (cm)		Step time *(ICC)*	95% CI *min*, *max* (s)	
		IMU system	Motion capture system		IMU system	Motion capture system
Participant 1	0.731^*^	16.88–17.21	16.96–17.35	0.731^*^	0.51–0.52	0.51–0.52
Participant 2	0.821^*^	33.27–33.75	31.70–32.14	0.820^*^	0.43–0.43	0.41–0.41
Participant 3	0.989^*^	7.88–8.31	7.85–8.31	0.989^*^	0.47–0.50	0.47–0.50
Participant 4	0.955^*^	17.09–17.43	17.09–17.45	0.955^*^	0.62–0.63	0.62–0.63
Participant 5	0.997^*^	15.57–16.94	14.94–16.29	0.997^*^	0.56–0.61	0.54–0.59
Participant 6	0.996^*^	14.83–15.90	14.94–16.00	0.996^*^	0.76–0.82	0.77–0.82
Participant 7	0.973^*^	6.88–7.19	6.82–7.14	0.973^*^	0.41–0.43	0.41–0.43
Participant 8	0.874^*^	18.53–19.02	17.19–17.72	0.873^*^	0.51–0.53	0.48–0.49
Participant 9	0.758^*^	15.11–15.34	15.22–15.45	0.758^*^	0.51–0.52	0.51–0.52
Participant 10	0.772^*^	31.57–31.98	31.47–31.83	0.772^*^	0.57–0.58	0.57–0.57
Participant 11	0.924^*^	17.90–18.20	18.25–18.41	0.924^*^	0.54–0.55	0.54–0.55
Participant 12	0.902^*^	59.39–60.16	59.34–60.17	0.901^*^	0.76–0.77	0.76–0.77
Participant 13	0.928^*^	21.39–21.79	21.62–22.00	0.927^*^	0.70–0.71	0.71–0.72
Participant 14	0.957^*^	8.34–8.53	8.36–8.54	0.957^*^	0.43–0.44	0.43–0.44
Participant 15	0.993^*^	22.98–24.19	23.25–24.48	0.993^*^	0.64–0.67	0.64–0.68
Participant 16	0.988^*^	32.10–32.99	32.18–33.07	0.988^*^	0.68–0.70	0.68–0.70
Participant 17	0.988^*^	38.12–39.46	37.91–39.27	0.988^*^	0.45–0.47	0.46–0.47

*results of the correlation analysis, *p* < 0.001; *CI* confidence interval, *ICC* intraclass correlation coefficient, *IMU* inertial measurement unit

## Discussion

The resultant linear acceleration, cadence, step length, and step time were hypothesized to show excellent agreement between the shoe-type IMU system and motion capture system for the 1-min treadmill walking by patients with PD. IMU systems have previously been compared with reference systems such as the motion capture system using healthy adults [11, 12, 14, 15], and the two systems have shown excellent agreement for the stride length, foot clearance, stride velocity, and turning angle (*r* range = 0.91–0.99) [11]; cadence (*ICC* = 0.998) and step length (*ICC* = 0.970) [12]; heel clearance, foot clearance, and foot angle (*r* range = 0.92–0.99) [14]; and linear accelerations along the anteroposterior, mediolateral, and vertical axes (*ICC* range = 0.75–0.94) [15]. Furthermore, several researchers have used participants with pathologies to determine the validity of IMU and reference systems such as the instrumented walkway system [5, 13, 16] and motion capture system [7, 17]. The step time (*ICC* = 0.981) and step length (*ICC* = 0.869) [5] and linear acceleration along the vertical axis (*ICC* > 0.75, *p* < 0.001) [7] showed good to excellent agreement, and the stride time [13, 16], step time (range = 4–9%) [13, 16], stride velocity, and stride length [25] showed low error values.

In the current study, the resultant linear accelerations for the left and right shoes indicated excellent agreement between the shoe-type IMU system and motion capture system. Furthermore, the resultant linear accelerations for each participant with PD indicated excellent agreement between the two systems for both the left (*ICC* range = 0.961–0.985) and right (*ICC* range = 0.944–0.982) shoes. In addition, the cadence, left step length, right step length, left step time, and right step time indicated excellent agreement between the two systems. This is similar to the results obtained in previous studies [5, 7, 11, 12, 14–17]. Furthermore, previous studies have reported an RMSE for the linear accelerations along the vertical axis for the two systems of 1.21 ± 1.11 m s^− 2^ (10.2 ± 9.3%), which is relatively small [7]. In this study, the RMSE values for the left and right shoes were 1.99 ± 0.63 m s^− 2^ (6.87 ± 1.56%) and 2.01 ± 0.58 m s^− 2^ (7.00 ± 1.39%), respectively. These are similar to the results of previous studies [7]. The resultant linear accelerations indicated that most of the differences were between the upper and lower LOA for the left and right shoes (range = 93.02–94.01%). Therefore, these results suggest that shoe-type IMU systems can provide reliable data for gait analysis of participants with PD.

The foot is the initial segment that makes contact with the ground during walking, and the HS and TO events are defined as those when the foot makes contact with the ground and is taken off the ground, respectively [10, 12]. The shoe-type IMU system used in this study mounted sensors in the outsole of the shoe beneath the back of each foot.

This may be able to maintain stable sensor positions without hindering movement compared to the use of double-sided tape or Velcro straps. Furthermore, the advantages of this system include not only the ability to measure data on the left and right sides but also on both sides concurrently during gait-related tasks. These advantages make the estimation of HS and TO events more accurate [12]. To maximize these advantages of the shoe-type IMU system, data were not averaged by repeating several trials; instead, the real-time data of participants with PD were collected as they walked with a steady-state gait in a single direction for 1 min for analysis. Thus, the results of this study may provide more meaningful and reliable data than previous methods. This may be useful for assessing characteristics related to pathologies such as asymmetry and variability during gait-related tasks. This requires the sensors to be attached to the lower limbs [5]. Therefore, the shoe-type IMU system may be useful for analyzing patients with PD because it may provide more reliable data during gait-related tasks in a clinical environment.

We determined that there was excellent agreement between the shoe-type IMU system and motion capture system during the 1-min treadmill walking by participants with PD, but there were several limitations for this study. First, 30 participants with PD were recruited, but only 17 performed the 1-min treadmill walking successfully. The causes of these results remain unclear; the gait characteristics of elderly adults may change during various walking speeds, or these results may have been affected by the relatively small sample size. If more participants had been recruited, then the agreement between the two systems may be more meaningful. In addition, the results were similar to those of previous studies that compared shoe-type IMU systems with motion capture systems for 1-min treadmill walking for young, middle-aged, and older adults [12]. Treadmill walking may be a useful task for a validity analysis of two systems because more steps under the steady-state gait condition can be acquired than with the participants walking along a walkway. However, this task may not be easy for participants with gait disturbances and more severe impairments (e.g., more than H&Y stage 3) to perform successfully. Some patients with PD indicated moderate *ICC* values (Range: 0.734–0.821) for the spatiotemporal parameters due to the delayed timing of HS events in the IMU system compared to the motion capture system, even when synchronizing the data between both two systems. These results may be related to the gait characteristics of patients with PD such as a tremor and shuffling steps; thus, these factors may affect the resultant linear acceleration in the IMU system. Finally, additional gait-related tasks such as turning, changing direction, and walking on irregular surfaces should be considered for gait analysis under realistic environmental conditions.

## Conclusions

In this study, the shoe-type IMU system and motion capture system exhibited excellent agreement for the resultant linear accelerations, cadence, step length, and step time. These results suggest that the shoe-type IMU system, which is relatively low in cost, small, and lightweight while providing reliable data, can be an alternative method for the gait analysis of patients with PD. Therefore, the shoe-type IMU system mounted on the outsole of shoes provides reliable data, and it may be useful for objective gait analysis of patients with PD in a clinical environment.

## Additional files

Additional file 1: IRB number. (PDF 60 kb)

Additional file 2: Raw data - resultant linear accelerations. (XLSX 9196 kb)

Additional file 3: Raw data - spatiotemporal parameters. (XLSX 158 kb)

Additional file 4: List of levodopa-equivalent dose. (XLSX 10 kb)

Additional file 5: Raw data - total resultant linear accelerations. (XLSX 15774 kb)

## Abbreviations

a: Acceleration
CI: Confidence intervals
COM: Center of mass
H&Y: Hoehn and Yahr stage
HS: Heel strike
*ICC*: Intraclass correlation coefficient
IMU: Inertia measurement unit
LOA: Limits of agreement
MC: Motion capture system
MDS-UPDRS: The modified movement disorder society version of the unified Parkinson’s disease rating scale
MMSE: Mini-mental state exam
PD: Parkinson’s disease
R: Resultant linear acceleration
RMS: Root mean square
RMSE: Root mean square error
TO: Toe off
