# Supplementary material for: Validity of shoe-type inertial measurement units for Parkinson’s disease patients during treadmill walking
Source: J Neuroeng Rehabil. 2018 May 15;15:38. doi: 10.1186/s12984-018-0384-9 (PMC5952468; doi:10.1186/s12984-018-0384-9)
Supplement: Supplementary file 1 — IRB number. (PDF 60 kb) [file 12984_2018_384_MOESM1_ESM.pdf]

결과통지서

2016년 9월 13일에 접수된 심의의견에 대한 답변서에 대하여 기관생명윤리위원회에서 심의하여 다음과 같이 결정하였음을 통지합니다.

<table>
<tr><td>과제번호</td><td colspan="3">2-104709-AB-N-01-201606-HR-025-04</td><td>관리번호</td><td>HR-025-04</td></tr>
<tr><td>연구과제명</td><td colspan="5">파킨슨병 환자의 보행로와 트레드밀 보행에 따른 보행특성</td></tr>
<tr><td>연구책임자</td><td>성명</td><td>이 명 곤</td><td>소속</td><td>대학원 건강과학과 운동역학</td><td>직위</td><td>박사과정생</td></tr>
</table>

<table>
<tr><td>심의대상</td><td colspan="3">☐ 연구계획서(신규) ■ 연구계획서(시정/보완)</td></tr>
<tr><td>심의일자</td><td>2016년 9월 19일</td><td>심의장소</td><td>동아대학교 기관생명윤리위원회</td></tr>
<tr><td>심의위원회</td><td colspan="3">동아대학교 기관생명윤리위원회</td></tr>
<tr><td>심의종류</td><td colspan="3">☐ 정규심의 ■ 신속심의</td></tr>
<tr><td>심의결과</td><td colspan="3">■ 승인 ☐ 시정승인 ☐ 보완 후 신속심의
☐ 보완 후 정규심의 ☐ 반려 ☐ 중지/보류</td></tr>
<tr><td>승인일자</td><td>2016.9.19.(월)</td><td>승인 유효기간</td><td>2017년 9월 18일</td></tr>
<tr><td>승인번호</td><td colspan="3">2-104709-AB-N-01-201606-HR-025-04</td></tr>
<tr><td>심의의견</td><td colspan="3">승인함</td></tr>
<tr><td>심의된 서류</td><td colspan="3">1. 심의의견에 대한 답변서
2. 변경대비표
3. 수정된 서류</td></tr>
</table>

본 통지서에 기재된 사항은 동아대학교 기관생명윤리위원회에 기록된 내용과 일치함을 증명합니다.
본 기관생명윤리위원회는 생명윤리 및 안전에 관한 법률과 관련 법규를 준수합니다.
본 연구와 이해상충(Conflict of Interest)이 있는 위원이 있을 경우 연구의 심의에서 배제하였습니다.
본 통지서의 사본은 기관생명윤리위원회에서 보관합니다.

※ 모든 연구자들은 아래의 사항을 준수하여야 합니다.

1) 승인된 계획서에 따라 연구를 수행하여야 합니다.
2) 위원회의 승인을 받은 동의서를 사용하여야 합니다.
3) 모국어가 한국어가 아닌 연구대상자들에게는 승인된 동의서를 연구대상자의 모국어로 인증된 번역본을 사용할 것이며 이러한 동의서 번역본은 반드시 위원회의 승인을 받아야 합니다.
4) 연구진행에 있어 연구대상자를 보호하기 위해 불가피한 경우를 제외하고 연구의 어떠한 변경이든 위원회의 사전 승인을 받고 수행하여야 하며 연구대상자들의 보호를 위해 취해진 어떠한 응급상황에서의 변경도 즉각 위원회에 보고하여야 합니다.
5) 위원회에서 승인된 계획서에 따라 등록된 어떠한 연구대상자라도 사망, 입원, 심각한 질병에 대하여는 위원회에 서면으로 보고하여야 합니다.
6) 연구 또는 연구대상자의 안전에 대해 유해한 영향을 미칠 수 있는 어떠한 새로운 정보도 즉각적으로 위원회에 보고하여야 합니다.
7) 위원회의 요구가 있을 때에는 연구의 진행과 관련된 보고를 위원회에 제출하여야 합니다.
8) 위원회가 심의한 과제에 대해 조사 및 감독 차원에서 현장점검을 실시할 시 원활한 점검절차 진행을 위해 연구자는 연구진행과 관련된 서류를 준비하고 협조하여야 합니다.
9) 연구대상자 모집광고를 사용할 시에는 사용 전에 위원회의 승인을 받아야 합니다.
10) 동의는 강제 혹은 부당한 영향이 없는 상태에서 충분한 설명에 근거하여 수행되어야 하며, 잠재적인 연구대상자에게 연구에 참여여부를 고려할 수 있도록 충분히 기회를 제공하여야 합니다.
11) 연구자와 그밖에 이해당사자는 연구계획서 승인을 광고나 홍보, 상업적 목적으로 사용할 수 없습니다.
12) 위원회의 심의결과 시정요구에 대해 모두 이행 및 충족될 경우에만 연구를 진행할 수 있습니다.
13) 위원회가 시정 및 보완을 요구한 경우 시정·보완 계획을 1개월 이내에 본 위원회에 제출하여야 합니다. 심의일로부터 1년 이내에 시정·보완 계획을 제출하지 않은 경우 심의가 무효화될 수 있습니다.
14) 시정계획은 신속심의로 진행되고 보완계획은 정규심의로 진행되며, 승인일과 승인 유효기간은 심의 결과에 따라 결정됩니다.
15) 승인기간 이후에도 연구를 지속하기 위해서는 적어도 승인 만료 2개월 전까지 연구의 진행상황에 대하여 중간보고를 하여야 합니다.
16) 연구 종료 후 3개월 이내에 종료보고를 하여야 합니다.
17) 연구와 관련된 기록은 연구가 종료된 시점을 기준으로 최소 3년간 보관하여야 합니다.

2016년 9월 19일

동아대학교 기관생명윤리위원회위원장(인)

동아대학교 기관생명윤리위원회위원장

본 통지서에 기재된 사항은 동아대학교 기관생명윤리위원회에 기록된 내용과 일치함을 증명합니다.
본 기관생명윤리위원회는 생명윤리 및 안전에 관한 법률과 관련 법규를 준수합니다.
본 연구와 이해상충(Conflict of Interest)이 있는 위원이 있을 경우 연구의 심의에서 배제하였습니다.
본 통지서의 사본은 기관생명윤리위원회에서 보관합니다.
